# Evaluation of bias and gender/racial concordance based on sentiment analysis of narrative evaluations of clinical clerkships using natural language processing

**DOI:** 10.1186/s12909-024-05271-y

**Published:** 2024-03-15

**Authors:** Sonali Bhanvadia, Bharanidharan Radha Saseendrakumar, Joy Guo, Maxwell Spadafore, Michelle Daniel, Lina Lander, Sally L. Baxter

**Affiliations:** 1 Division of Ophthalmology Informatics and Data Science, Viterbi Family Department of Ophthalmology and Shiley Eye Institute, University of California San Diego, La Jolla, CA USA; 2 Health Department of Biomedical Informatics, University of California San Diego, La Jolla, CA USA; 3 Department of Emergency Medicine, University of Michigan Medical School, Ann Arbor, MI USA; 4 Department of Emergency Medicine, University of California San Diego, La Jolla, CA USA; 5 Department of Family Medicine and Public Health, University of California San Diego, La Jolla, CA USA

**Keywords:** Natural language processing, Bias in medical education, Medical student evaluations, Narrative evaluations

## Abstract

There is increasing interest in understanding potential bias in medical education. We used natural language processing (NLP) to evaluate potential bias in clinical clerkship evaluations. Data from medical evaluations and administrative databases for medical students enrolled in third-year clinical clerkship rotations across two academic years. We collected demographic information of students and faculty evaluators to determine gender/racial concordance (i.e., whether the student and faculty identified with the same demographic). We used a multinomial log-linear model for final clerkship grades, using predictors such as numerical evaluation scores, gender/racial concordance, and sentiment scores of narrative evaluations using the SentimentIntensityAnalyzer tool in Python. 2037 evaluations from 198 students were analyzed. Statistical significance was defined as *P* < 0.05. Sentiment scores for evaluations did not vary significantly by student gender, race, or ethnicity (*P* = 0.88, 0.64, and 0.06, respectively). Word choices were similar across faculty and student demographic groups. Modeling showed narrative evaluation sentiment scores were not predictive of an honors grade (odds ratio [OR] 1.23, *P* = 0.58). Numerical evaluation average (OR 1.45, *P* < 0.001) and gender concordance between faculty and student (OR 1.32, *P* = 0.049) were significant predictors of receiving honors. The lack of disparities in narrative text in our study contrasts with prior findings from other institutions. Ongoing efforts include comparative analyses with other institutions to understand what institutional factors may contribute to bias. NLP enables a systematic approach for investigating bias. The insights gained from the lack of association between word choices, sentiment scores, and final grades show potential opportunities to improve feedback processes for students.

**Supplementary Information:**

The online version contains supplementary material available at 10.1186/s12909-024-05271-y.

## Introduction

Considerations of systemic and institutional racism have become more prominent with the Black Lives Matter movement and other national events [1]. There is increasing interest in understanding whether bias is present in medical education [2]. Determination of bias in medical education is the first step in promoting equity and diversity as it allows for awareness that can be used to inform downstream interventions to address the issue [3]. Through this, introduction of different learning programs, such as cultural competence training, can be developed for medical education programs [4]. Equity in education and healthcare is needed to provide the same opportunities to all members and results in more positive patient outcomes [5]. Bias in medical training can negatively affect diversification efforts, which is important in ensuring all backgrounds and beliefs are equally represented in medicine [6, 7]. 

Prior studies have demonstrated potential disparities in the words used to describe medical students in their evaluations, such as variations by gender or by race [8, 9]. Natural language processing (NLP) has previously been used to determine narrative differences between medical clerkship evaluations based on gender and under-represented minority status [8]. It has been found that the most commonly used words for females and males differ. Females are described using descriptive words for personal attributes more often than males, who are more likely to be described using words that explain their competency and behaviors [8]. Similar conclusions have been made when clerkship evaluations are examined by word choice, in which results show that females and minorities are described differently than males and White medical students [9]. Narrative evaluations are extremely important for medical students, especially given that narrative evaluations are part of their residency application and can play a key role in their career potential and residency acceptances [10]. 

In this study, we leveraged NLP techniques to analyze core clerkship evaluations to investigate whether sentiment or word choice varied significantly by race/ethnicity or gender. We aimed to evaluate the presence of bias among narrative core clinical clerkship evaluations of third-year medical students and whether variations in clerkship evaluation text were associated with overall clerkship grades, with a secondary aim of evaluating whether potential bias was associated with gender or racial concordance between evaluating faculty members and students. This addresses an important gap in the literature regarding potential bias in narrative evaluations and the role of both gender and racial concordance between faculty and students, which has not been previously well-studied.

## Methods

### Study population

This study was conducted at the University of California San Diego (UCSD) School of Medicine, an accredited allopathic medical school located in La Jolla, CA. Data were extracted from medical evaluation databases for medical students enrolled in third-year core clinical clerkship rotations across two academic years (2019–2020 and 2020–2021). These clerkships included medicine, neurology, reproductive medicine, pediatrics, psychiatry, and surgery. The UCSD Institutional Review Board (IRB) approved this study.

**Fig. 1:**
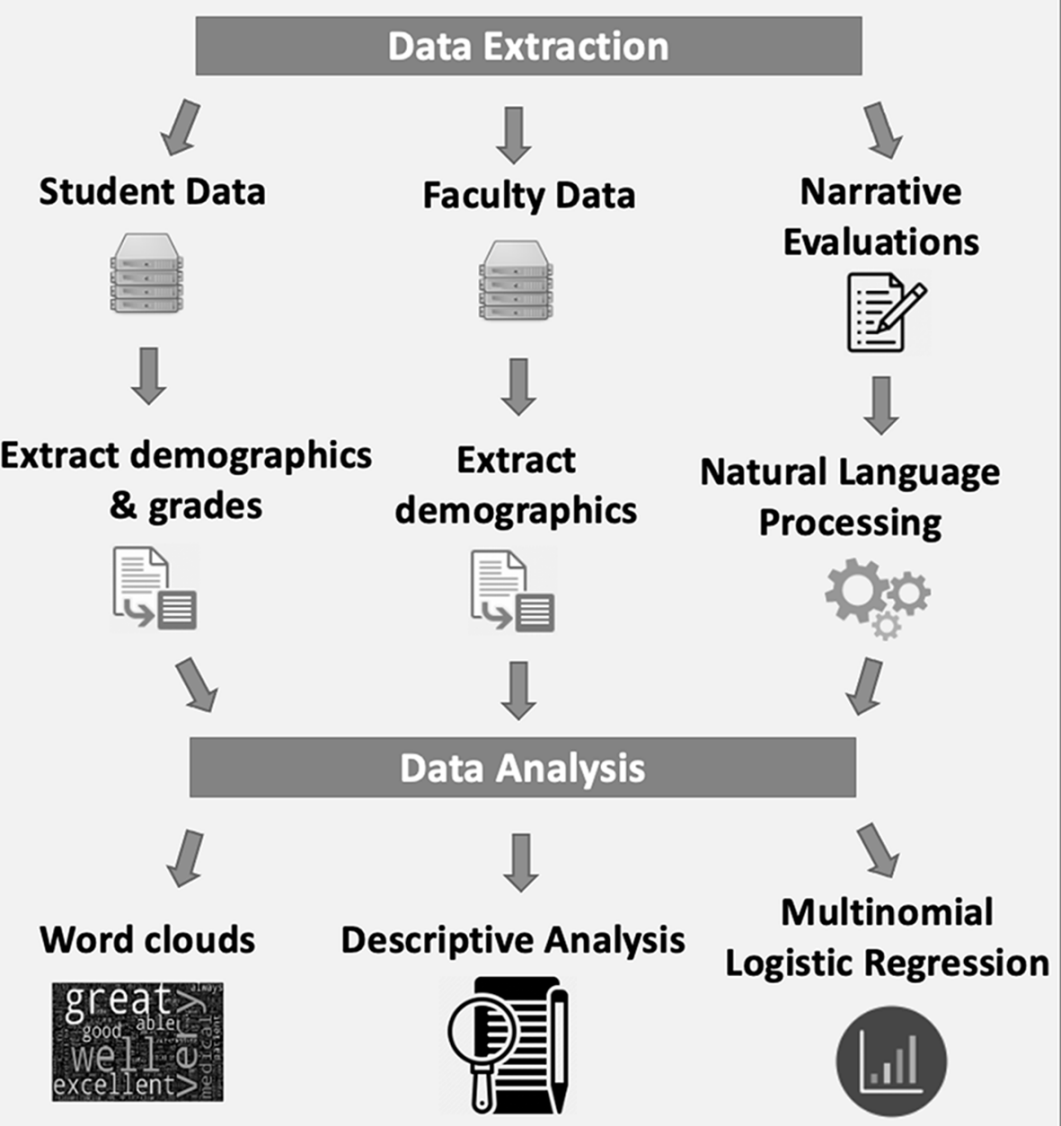
Overall study workflow

### Data sources

Data were extracted by administrators at the UCSD School of Medicine. Student and faculty demographics, such as their race, ethnicity, gender, as well as administrative/academic data such as clerkship/specialty (for evaluators), grades received (for students), and academic year/term, were collected (Fig. 1). Only students who had faculty evaluators with available demographic data were included. For example, some faculty evaluators were not UCSD School of Medicine faculty and were based at outside hospitals where students rotated during their clerkships, but because these evaluators did not have demographic data available in UCSD databases (and thus racial and gender concordance could not be determined), these evaluations were not provided to the research team and therefore were not included in the analysis. Narrative evaluations were also collected. This consisted of extraction of the complete full text of each evaluation in preparation for downstream analyses, which may have included non-Medical Student Performance Evaluation (MSPE) comment data within the full free-text evaluations. Other academic data that were extracted included numerical evaluation grades from each faculty member (graded on a 7-point competency scale) and the overall final clerkship grades (Pass, Near Honors, or Honors), in which the NBME shelf exam scores are included.

### Data cleaning/processing

Race and ethnicity mapping were necessary to clean the demographic data, in which both race and ethnicity demographics for students and faculty were harmonized, since the original source data for faculty were more specific/granular than those for students (e.g., faculty demographic data listed specific countries for race/ethnicity categories). Race was categorized by the following: White, Black or African American, American Indian or Alaska Native, Asian, Native Hawaiian or Other Pacific Islander, and Two or More Races. Ethnicity was categorized as Hispanic or Latino, Not Hispanic or Latino, and Prefer Not to Answer/Unknown. These categories are those recognized by the U.S. Census Bureau [11, 12].

Variables for concordance were defined by comparing the demographic characteristics of the students with the demographic characteristics of their evaluators. If a given student and a given faculty member self-reported the same race, same gender, or the same ethnicity, they would be recorded as “concordant” for that feature. Race concordance, gender concordance, and ethnicity concordance were therefore secondarily derived and included in subsequent analyses as covariates.

### Natural language processing

Natural language processing (NLP) was performed on the unstructured/narrative free-text of the clerkship evaluations of included students. In preparation for sentiment analysis, stop words (such as *the*, *is*, *at, which*, or *on*) and numbers were removed from the evaluations, and words were lemmatized using modules from the *nltk* python package [13]. Lemmatization aggregates different forms of a word together and categorizes them as a “base” word or lemma (e.g., “talk” and “talking” can be lemmatized to “talk”). The processed evaluations were subsequently fed through the VADER sentiment analyzer, a rule-based sentiment analysis model [14]. The analyzer assigns a sentiment score for each comment ranging from − 1.0 (extremely negative) to 1.0 (extremely positive). The sentiment score was generated by the SentimentIntensityAnalyzer from the Natural Language Toolkit (NLTK) package in Python, based on the VADER sentiment analysis tool [15]. This analyzer assigns a compound score to each sentence in the evaluation to provide the overall evaluation sentiment score. Although VADER was originally trained with short sentences and phrases, this approach of using it in conjunction with NLTK has been shown to be accurate when used on longer text as well [16]. We used these sentiment scores to represent the overall positivity or negativity of each student evaluation. Examples of narrative evaluations and their corresponding sentiment scores are provided in Supplemental Table X.

For a more qualitative analysis of word choice used in medical student clerkship evaluations, word clouds were generated using the *wordcloud* Python package [17]. Comparative word clouds were generated by race, ethnicity, and gender of students. The number of words used to generate word clouds were, at most, the top 1000 adjectives and adverbs used in the narrative evaluations.

Additional quantification of variations in word choice was performed by using conditional probabilities to generate likelihood ratios for different words used for different categories of students. This approach allows identification of words with the greatest differential use across categories, rather than focusing on the most common words within a category. For example, for analysis by gender, likelihood ratios were calculated to identify words that had the greatest amount of difference in usage among evaluations of male students compared to female students. Words used for this analysis were token words, which were adjectives and adverbs found in at least 1.5% of all written evaluations. It is customary in natural language processing to remove very high-frequency words (i.e. “stop words” such as “a,” “an,” and “the”) as well as very low-frequency words. Here, we chose > 1.5% as the threshold based on empiric evaluation of the data, as any words found in a lower than 1.5% frequency within all the evaluations would not have been beneficial to the analysis, as they were not mentioned often enough. These likelihood ratios were also analyzed by race and ethnicity.

### Statistical analyses

Descriptive analyses were conducted to compare demographic characteristics among the medical students and the evaluating faculty, as well as distributions of clerkship grades and sentiment scores of narrative evaluations. Grade distributions were analyzed by gender, race, and ethnicity using the Chi-Squared test, with *p* < 0.05 as the threshold for statistical significance. Sentiment score distributions were also analyzed by race, gender, ethnicity, term, and clerkship with the use of Student’s t-tests after verifying that assumptions necessary for parametric hypothesis testing were met. Multiple analysis of variance (ANOVA) was performed to compare how final letter grades varied by clerkship evaluation sentiment scores.

T-tests and chi-squared tests were used to compare between continuous variables and categorical variables, respectively. The t-test was performed for comparing scores between groups for each variable: Race_Concordance (i.e., mean sentiment score among faculty/student pairs with concordant race vs. mean sentiment score among faculty/student pairs without concordant race), Ethnicity_Concordance (i.e., mean sentiment score among faculty/student pairs with concordant ethnicity vs. mean sentiment score among faculty/student pairs without concordant ethnicity), and Gender_Concordance (i.e., mean sentiment score among faculty/student pairs with concordant gender vs. mean sentiment score among faculty/student pairs without concordant gender).

Multinomial logistic regression (log-linear) modeling via neural networks from the *nnet* package in R was used to predict the primary outcome of interest, which was the overall clerkship evaluation grade received [18]. A grade of “Pass” was used as the reference group, and models were generated to examine the odds of Near Honors and the odds of Honors based on various factors, such as numerical evaluation scores (which were averaged), gender concordance, race concordance, and narrative evaluation sentiment score. A multinomial log-linear model was used instead of an ordinal regression model because ordinal regression models typically assume proportional odds from one level to the next. A majority of students had 1–2 evaluations per clerkship/letter grade pair. This multinomial log-linear model could not accommodate mixed effects, and while generalized estimating equations (GEEs) were initially considered to account for potential clustering effects, we ultimately did not use them because GEEs are typically intended for simple clustering or repeated measures, whereas the combination of characteristics from students, faculty, demographic concordance features, and grades generated a high level of complexity. Because our primary objective was to understand the potential effects of sentiment attached to free-text evaluations, we modeled at the level of the evaluation to enhance model interpretability.

Data wrangling and sentiment score analysis were done in Python version 3.9, and other statistical analyses (descriptive analyses and regression modeling) were performed using R 4.2.1.

We calculated likelihood ratios for all words (w) that were adjectives and adverbs and appeared in at least 1.5% of all narrative evaluations. A total of 102 token words were found among all narrative evaluations. Partitioning was completed by determining which evaluations were specific to some demographic factor, such as gender (i.e. M_male_ was labeled for evaluations that were specific to males, and M_female_ was used for evaluations specific to females). Conditional probabilities showed the frequency of a token word appearing for a specific demographic group’s evaluations. Then, likelihood ratios were calculated to quantify the number of times more likely a word was appearing in an evaluation of a specific demographic group in comparison with another. As an illustration, to evaluate differential word choice by gender, the following formula was used to calculate the likelihood ratio (*LR*) for a given token word *w* based on conditional probabilities (*P*) of *w* based on frequencies (*f*) of usage in the evaluations of males and females, respectively:

$$\eqalign{ LRw{\rm{ }} = & {\rm{ }}(P(w|male))/(P(w|female)) \cr & = {\rm{ }}(f(Mmale, w))/(f(Mfemale,w)) \cr} $$

The log(LR) of token words in each partitioned sub-matrix was visualized using the *matplotlib* library in Python version 3.9. Insubstantial log(LR) values between − 0.05 and 0.05 were omitted from likelihood graphs for ease of interpretation.

## Results

### Study population characteristics

**Table 1:** Demographics of students and evaluators involved in core clerkship rotations

Demographics		
	Students (*N* = 198)	Evaluators (*N* = 196)
Race		
Asian	77(39%)	53(27%)
Black/African American	13(7%)	5(3%)
White	92(46%)	102(52%)
Other	13(7%)	32(16%)
Ethnicity		
Hispanic or Latino	10(5%)	7(4%)
Not Hispanic or Latino	185(93%)	185(94%)
Gender		
Female	109(55%)	98(50%)
Male	89(45%)	92(46%)

There were a total of 196 evaluators and 198 students. Demographic characteristics for both groups are detailed in Table 1. Slightly more than half (109/198, 55%) of students identified as females. Approximately half (98/196, 50%) of the evaluators identified as females. Regarding racial composition, Whites were most well-represented among both students and evaluators, comprising approximately half of each group (Table 1). Underrepresented minorities had greater representation among the students compared to the faculty evaluators (7% vs. 3% Black and 5% vs. 4% Hispanic/Latino; Table 1).

### Sentiment scores of narrative evaluations

2037 narrative evaluations were analyzed, in which sentiment scores were computationally determined in a range from − 1.0 (extremely negative) to 1.0 (extremely positive), with 0 being neutral. The mean (SD) sentiment score was 0.3 (0.2). The sentiment scores of the evaluations did not vary significantly by student gender, race, or ethnicity (*P* = 0.88, 0.64, and 0.06, respectively) (Table 2). Only 41 (2.01%) of the narrative evaluations were found to have negative sentiment scores. A qualitative assessment of these evaluations revealed that they contained phrases such as “Limited interaction”, “Inadequate contact”, “Insufficient exposure to the student”, etc. that contributed to their negative scoring.

**Table 2:** Sentiment scores based on evaluator and student demographics

Evaluator	Student	Mean (SD) sentiment score	p-value
**Gender** ^**a**^			
Female	Female	0.29(0.22)	0.88
	Male	0.29(0.21)	
Male	Female	0.32(0.25)	
	Male	0.33(0.24)	
**Race** ^**b**^			
Asian	Asian	0.31(0.22)	0.64
	African American	0.33(0.23)	
	White	0.33(0.24)	
	Other	0.30(0.19)	
African American	Asian	0.35(0.10)	
	African American	0.19(0.09)	
	White	0.27(0.15)	
	Other	0.25(0.01)	
White	Asian	0.31(0.24)	
	African American	0.24(0.27)	
	White	0.29(0.23)	
	Other	0.33(0.23)	
Other	Asian	0.29(0.22)	
	African American	0.19(0.22)	
	White	0.26(0.20)	
	Other	0.24(0.16)	
**Ethnicity** ^**c**^			
Hispanic or Latino	Hispanic or Latino	0.28(0.20)	0.06
	Not Hispanic or Latino	0.31(0.23)	
Not Hispanic or Latino	Hispanic or Latino	0.30(0.21)	
	Not Hispanic or Latino	0.30(0.23)	

^a^ Gender_Concordance (i.e., mean sentiment score among faculty/student pairs with concordant gender vs. mean sentiment score among faculty/student pairs without concordant gender)

^b^ Race_Concordance (i.e., mean sentiment score among faculty/student pairs with concordant race vs. mean sentiment score among faculty/student pairs without concordant race)

^c^ Ethnicity_Concordance (i.e., mean sentiment score among faculty/student pairs with concordant ethnicity vs. mean sentiment score among faculty/student pairs without concordant ethnicity)

### Clerkship grade distributions

**Table 3:** Distribution of core clerkship grades by race and gender. *N = 418 as some students may have multiple grades, due to there being more than one term/clerkship for which they had evaluations

Overall Grade Distributions					
*N* = 418*	Honors	Near Honors	Pass	Total	p-value
By Race (N, %)					
African American	4(16%)	9(36%)	12(48%)	25	*p* = 0.63
Asian	49(30%)	49(30%)	67(60%)	165	
White	71(36%)	59(30%)	67(34%)	197	
Other	8(26%)	8(26%)	15(48%)	31	
By Gender (N, %)					
Female	80(34%)	70(29%)	88(37%)	238	*p* = 0.57
Male	52(29%)	55(31%)	73(41%)	180	
Numerical Evaluation Average (Mean(Standard Deviation) on a 7-point scale)	5.9 (1.0)	5.8 (1.0)	5.4 (1.2)		*p* < 0.001

Grade distributions by race and gender are shown in Table 3. Females (80/238, 34%) were more likely to receive honors than males (52/180/420, 29%; *p* = 0.57). More students received an honors grade during the 2020–2021 term (174/418, 42%) than during the 2019–2020 term (123/510, 24%; *p* < 0.001). Students who received higher numeric evaluation averages were more likely to receive higher overall clerkship grades than those who received lower numeric evaluation averages (*p* < 0.001).

### Models of overall clinical clerkship grades

The multinomial log-linear model of final overall clerkship grades showed that the narrative evaluation sentiment score was not predictive of an honors grade (odds ratio [OR] 1.61 [95% Confidence Interval, CI, 0.75–3.45], *P* = 0.23) nor a near honors grade (OR 2.06 [0.90–4.72], *p* = 0.09; Table 4). However, the numerical evaluation average was significantly associated with higher odds of receiving honors or near honors (OR 1.53 and OR 1.43, respectively, both *P* < 0.001; Table 4). Gender concordance between faculty and student had a borderline significant association with receiving honors (OR 1.32, *P* = 0.053, Table 4) but not near honors. Race concordance between faculty and students was not significantly associated with the overall clerkship grade.

**Table 4:** Multinomial log-linear model of overall clerkship grades

Variable	Honors			Near Honors		
	Adjusted OR	95% CI	p-value	Adjusted OR	95% CI	p-value
(Intercept)	0.035	(0.02, 0.08)	< 0.001 ***	0.04	(0.02, 1.00)	< 0.001 ***
Numerical Evaluation Average	1.53	(1.34, 1.75)	< 0.001 ***	1.43	(1.24, 1.66)	< 0.001 ***
Gender Concordance	1.32	(1.00, 1.74)	0.053 *	1.20	(0.89, 1.63)	0.24
Race Concordance	1.12	(0.84, 1.50)	0.426	1.15	(0.84, 1.6)	0.001***
Term/Academic Year	2.86	(2.15, 3.80)	0.001***	2.38	(1.74, 3.24)	0.001***
Sentiment Score of Free-Text Narrative Comments	1.61	(0.75, 3.45)	0.23	2.06	(0.90, 4.72)	0.09

### Word clouds

**Fig. 2:**
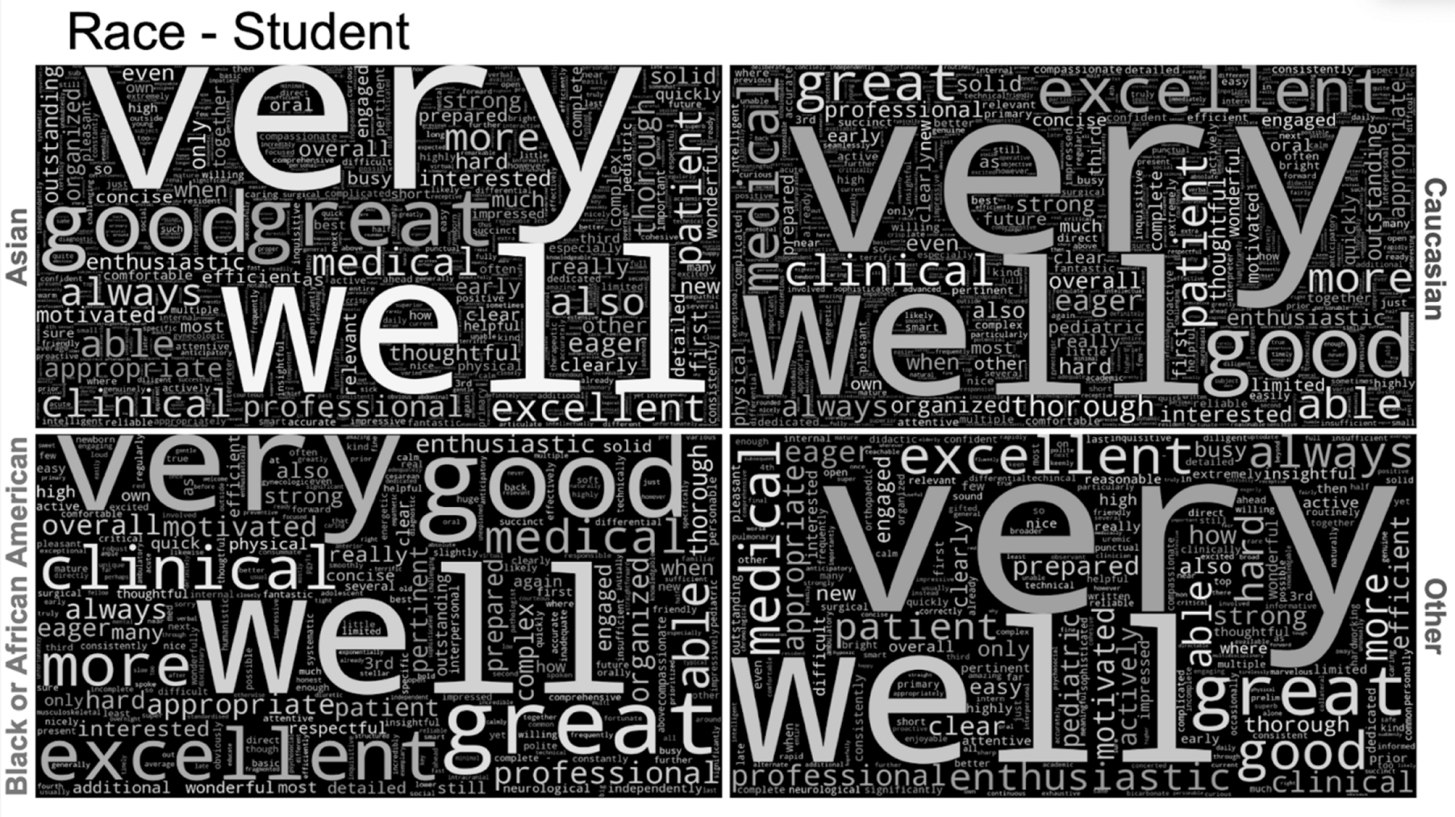
Word clouds demonstrating word choice in narrative evaluations by race

Generated word clouds showed that word choice in narrative evaluations were found to be very similar for all students, even when compared by race, gender, ethnicity, and evaluator demographics. Some of the most commonly used words amongst all groups were: *well*, *great*, *good*, *excellent*, *enthusiastic*, *thorough*, *professional*, *interested*, *strong*, *concise*, *clinical*, *engaged*, and additional similar adjectives (Table 5). Word clouds show the similarities amongst commonly used words for students of different racial background (Fig. 2). Word clouds by other demographic groups looked similar. Narrative evaluations were also found to have similar word choice regardless of grades received.

**Table 5:** Most commonly used words in narrative evaluations, in decreasing frequency of use

Most Commonly Used Words		
**Gender of Student**		
Female	Male	
Very, well, great, good, excellent, enthusiastic, thorough, thoughtful, professional, outstanding, strong, always, clinical, medical, patient, eager, interested, appropriate, engaged, concise, hard	Very, well, good, excellent, great, patient, medical, professional, eager, more, always, enthusiastic, motivated, thorough, strong, organized, interested, thoughtful, clinical	
**Race of Student**		
African American	White	Asian
Very, good, well, great, excellent, able, clinical, enthusiastic, prepared, pertinent, professional, interested, thorough, engaged, complex, appropriate, interpersonal, respectful, solid, detailed, organized, wonderful, patient	Very, good, excellent, well, always, strong, medical, clinical, professional, thorough, great, patient, prepared, concise, enthusiastic, eager, thoughtful, engaged, kind, attentive, outstanding, interested, motivated, appropriate	Very, well, good, clinical, excellent, great, thoughtful, appropriate, eager, concise, outstanding, thorough, professional, enthusiastic, organized, prepared, relevant, engaged, medical, strong, clear, able, wonderful
**Letter Grade Received**		
Pass	Near Honors	Honors
Very, well, excellent, able, good, great, professional, medical, appropriate, thorough, clinical, enthusiastic, quick, motivated, patient, engaged, solid	Very, well, good, excellent, more, interested, eager, concise, always, medical, patient, pleasant, professional, clinical, appropriate, efficient, strong, engaged, able, organized, impressive	Very, well, outstanding, clinical, thorough, patient, professional, good, great, excellent, medical, eager, able, wonderful, organized, detailed, appropriate, thoughtful, efficient, complex, quick, strong, concise

### Likelihood ratios

A total of 102 token words (adjectives and adverbs that appeared in at least 1.5% of all narrative evaluations) were found amongst all of the narrative evaluations.

For likelihood ratios, we compared the likelihood of specific terms being used amongst the following groups: males versus females, Hispanic/Latino students versus Non-Hispanic/Latino students, White students versus all other race groups, and Asian students versus all other race groups. A majority of words used to describe all groups were found to have positive sentiment, or were words considered to be of positive regard. Positive words, such as *good, great, excellent, professional, outstanding, motivated, enthusiastic, strong, eager, thorough, clear, interested*, and similar words with positive sentiment were used across the board in students’ evaluations, regardless of race, gender, or grade received (Table 6). Words describing personality characteristics, such as *motivated, enthusiastic, strong, eager*, and others were commonly used for both females and males. Words describing professional competencies, such as *clinical, professional, medical, appropriate*, and other words, were used for everyone, regardless of what demographics they identified as.

**Table 6:** Likelihood ratios [LR] of token words by demographic groups

**More Likely for Male**		**More Likely for Female**	
**Word**	**LR**	**Word**	**LR**
smart	0.36	willing	1.71
much	0.37	active	1.642558
fantastic	0.43	how	1.49
confident	0.57	most	1.43
short	0.60	professional	1.40
wonderful	0.61	oral	1.38
complicated	0.62	patient	1.34
nice	0.62	clear	1.32
future	0.67	comfortable	1.30
complex	0.67	solid	1.30
**More Likely for Hispanic or Latino**		**More Likely for Not Hispanic or Latino**	
**Word**	**LR**	**Word**	**LR**
interested	0.24	easy	4.07
motivated	0.28	high	3.66
concise	0.39	confident	2.95
most	0.40	insightful	2.85
clinical	0.41	how	2.72
other	0.41	3rd	2.52
physical	0.47	actively	2.38
when	0.48	hard	2.21
oral	0.49	nice	2.19
own	0.50	pleasant	2.09
**More Likely for Asian**		**More Likely for Not Asian**	
**Word**	**LR**	**Word**	**LR**
limited	0.70	sure	2.07
3rd	0.72	only	1.86
future	0.73	important	1.82
kind	0.73	especially	1.81
complete	0.76	helpful	1.71
patient	0.77	impressed	1.57
clear	0.78	so	1.54
active	0.80	short	1.52
attentive	0.80	early	1.51
compassionate	0.80	new	1.46
**More Likely for White**		**More Likely for Not White**	
**Word**	**LR**	**Word**	**LR**
sure	0.36	future	1.72
insightful	0.39	complete	1.68
only	0.55	limited	1.58
easy	0.56	concise	1.47
important	0.57	strong	1.39
many	0.57	accurate	1.34
high	0.65	confident	1.33
impressed	0.68	just	1.33
helpful	0.69	third	1.33
efficient	0.70	patient	1.30

## Discussion

Prior studies have shown disparities in medical student evaluations by gender and race/ethnicity. In this study, we leveraged natural language processing techniques to analyze grades and narrative evaluations by student gender, race, and ethnicity, with an additional layer of analysis examining demographic concordance between student and faculty. This study aimed to determine if there was any gender, ethnic, or racial bias in the text of medical student evaluations and the potential influence on overall clerkship grades.

Our first key finding was that there was no clear evidence of textual bias in medical student evaluations in our sample at this institution. Overall, the overwhelming majority (98%) of evaluations were found to have positive sentiment. Only 2% of evaluations had negative sentiment based on the sentiment analysis, but closer examination of these evaluations demonstrated that they were not truly negative evaluations per se, but rather reflected a relative lack of contact with the student being evaluated. Sentiment scores from narrative evaluations were not significantly associated with final clerkship grades (Table 5). In other words, narrative feedback was found to be generally positive for all students regardless of final grade they would receive. Although prior studies have shown a tendency toward personal qualities being highlighted in evaluations of female students vs. professional qualities being highlighted in male students [8, 9], we did not find clear differences in the types of words used by gender, race, or ethnicity. The lack of disparities in the narrative text examined in our study contradicts the results of previous studies at other institutions. These may be in part explained by geographical variation, as our study was conducted at a public institution with a fairly diverse student population. Additionally, because many of these narrative evaluations are from a more recent time period, there may be increased awareness in regards to racial bias. Since The Black Lives Matter movement began, there has been an increase in anti-racism and anti-bias training in medical education [19, 20]. Similarly, the COVID-19 pandemic brought to light disparities in medical treatment and education, which has also triggered an increase in diversity, equity, and inclusion (DEI) training for students and faculty [21]. 

Another key finding was that although narrative evaluation sentiment scores were not predictive of an honors grade, the numerical evaluation averages were significantly associated with clerkship grades. Numerical evaluation average was directly correlated with the odds of receiving an honors or near honors grade. These numerical evaluations were Likert scale scores provided by faculty, and their correlation with clerkship grades demonstrated that these may be more reflective of the faculty’s assessment of the student’s clinical performance compared to the narrative feedback, which generally was positive regardless of the final grade. This may have in part simply reflected the process of calculating the clerkship grades, and we could not consistently analyze this across the cohort due to variations in individual clerkship grading policies during the study period. Additionally, we acknowledge that this finding may not necessarily generalize to other institutions. However, the finding that narrative evaluations tend to not correlate well with final grades is still worth noting. Transitioning toward more milestone-based competencies may be a more objective and informative evaluation approach compared to narrative evaluations. This is an approach being increasingly used by the Accreditation Council of Graduate Medical Education, [22] and may deserve greater consideration in the undergraduate medical education setting.

Gender concordance was another factor which was found to have a borderline association with receiving an honors grade (OR 1.32, *P* = 0.053). Although it is a borderline finding, it could show that there may be some tendency for evaluators to give better narrative evaluations to students who identify as the same gender as themselves. Future investigations with increasing sample sizes are needed to assess whether the association between gender concordance and grades is replicated. This inclusion of the role of demographic concordance between medical students and faculty evaluators is a unique strength of this study, as it has not been well-studied previously.

We inferred that there may have been a lack of formative feedback helpful to the students for improvement, since students who did not receive honors or near honors also had fairly positive narrative evaluations. This finding highlights the need to better understand how narrative evaluations can be improved, as formative feedback is important for students to have for the purpose of learning. Constructive feedback is extremely beneficial in medical education, as it allows students to recognize where they can improve, which in turn allows them to become stronger healthcare providers [23]. Feedback is a pillar in medical education and one of the most important factors of the learning experience [24]. 

Students have been found to appreciate and seek honest feedback from their mentors and evaluators, regardless of whether the feedback is negative or positive, as they find it necessary to their learning journey [25, 26]. Mentors and evaluators may find it difficult to give feedback to students as they do not want to cause harm to their professional careers, and know the weight that narrative evaluations can hold for students [27, 28]. Providing venues for providing formative feedback that are not associated with a formal grade or evaluation may be one strategy for addressing this issue. Another potential approach is to pursue peer feedback. Training peers to be good evaluators, and to help give constructive criticism to their peers, helps students not only make improvements, but also allows those giving the peer evaluations the training to provide this feedback to students when they become physicians and mentors [26]. 

### Limitations

One limitation of evaluating the narrative evaluations with NLP is that a majority of words in narrative evaluations tend to be what VADER labels as “positive” in sentiment. Therefore, words such as “good” and “okay” would be considered to be “positive”, although they are often not reflective of positive ratings when considered in context of medical evaluations, whereas words such as “excellent” and “outstanding” are. Due to this, narrative evaluations may be considered to be more positive in sentiment with the use of NLP, although they may not be when comparatively analyzing one evaluation against another. Similarly, the sentiment analyzer we used provided a general sentiment score but did not have capability to distinguish text that was purely praise from text that had actionable components; this would be an interesting avenue for future research. In addition, future studies could utilize more advanced techniques such as attention-based transformer networks, such as those used in large language models.

For this study, we did not look at NBME/shelf exam scores, which were also factored into the grades that students received, but exactly how shelf exam scores were incorporated varied by year and by individual clerkships. Therefore, we were unable to systematically incorporate shelf exam scores into our analysis. Furthermore, the main focus of this study was on the narrative text evaluations, since that has been less well-explored in prior literature.

Another limitation is that grades during this time period, due to the COVID-19 pandemic, may have been inflated. We had nearly double the number of honors grades in the 2020–2021 academic year in comparison to the 2019–2020 year in our dataset. Studies have shown that during the pandemic, grades for students enrolled in higher education had high levels of inflation, and there were less variations in grades [29]. Although, there are also various studies that also show some students did perform worse than previous semesters, due to changes in study habits, mental health, and changes in student/faculty relationships due to remote learning [30]. 

We were also unable to include student evaluations from prior to the COVID-19 pandemic, so we did not have the benefit of historical control that pre-COVID grades and evaluations would have provided. During the pandemic, the medical evaluation platform at this institution changed. Data extraction from the older system was proven to be difficult, especially for the unstructured data that was needed for this analysis. Therefore, this analysis was focused on the newer evaluation system. Future studies may benefit from using pre-pandemic data for further comparison.

## Conclusion

There was limited evidence of bias in medical student narrative text evaluations at this institution based on analysis of demographics and using NLP for sentiment and word choice analysis, which contrasts some prior published studies. Ongoing investigations to understand these contrasting findings include comparative analyses with data from other institutions that may vary in type (public vs. private), student body, or location. Formative feedback is beneficial for students, especially in the healthcare setting, and the lack of association between textual analysis of narrative evaluations and overall clerkship grades indicates a potential gap in feedback for medical students. Future studies may investigate how to provide constructive feedback while continuing to mitigate any potential bias.

### Electronic supplementary material

Below is the link to the electronic supplementary material.

Supplementary Material 1

## Data Availability

The data that support the findings of this study are available from University of California, San Diego School of Medicine but restrictions apply to the availability of these data, which were used under license for the current study, and so are not publicly available. Data are however available from the authors upon reasonable request and with permission of University of California, San Diego School of Medicine.

## References

[1] Jee-Lyn García J, Sharif MZ (2015). Black lives matter: a commentary on racism and Public Health. Am J Public Health.

[2] FitzGerald C, Hurst S (2017). Implicit bias in healthcare professionals: a systematic review. BMC Med Ethics.

[3] Javier D, Solis L, Paul MF (2022). Implementation of an unconscious bias course for the National Research Mentoring Network. BMC Med Educ.

[4] Amutah C, Greenidge K, Mante A (2021). Misrepresenting race - the role of medical schools in propagating Physician Bias. N Engl J Med.

[5] Williams JS, Walker RJ, Egede LE (2016). Achieving equity in an Evolving Healthcare System: opportunities and challenges. Am J Med Sci.

[6] Stanford FC (2020). The importance of diversity and inclusion in the Healthcare workforce. J Natl Med Assoc.

[7] Jasmine R, Marcelin, Dawd S, Siraj R, Victor S, Kotadia YA, Maldonado. The impact of unconscious Bias in Healthcare: how to recognize and mitigate it. J Infect Dis, 220, Issue Supplement_2, 15 September 2019, Pages S62–S73, 10.1093/infdis/jiz214. 10.1093/infdis/jiz214 31430386

[8] Rojek AE, Khanna R, Yim JWL (2019). Differences in Narrative Language in evaluations of medical students by gender and under-represented minority status. J GEN INTERN MED.

[9] Axelson RD, Solow CM, Ferguson KJ, Cohen MB (2010). Assessing implicit gender Bias in Medical Student performance evaluations. Eval Health Prof.

[10] Buchanan AO, Strano-Paul L, Saudek K (2022). Preparing effective narrative evaluations for the Medical School performance evaluation (MSPE). MedEdPORTAL.

[11] https://www.census.gov/quickfacts/fact/note/US/RHI625221.

[12] https://www.census.gov/quickfacts/fact/note/US/RHI725221.

[13] Bird S, Edward Loper and Ewan Klein. Natural Language Processing with Python. O’Reilly Media Inc.; 2009.

[14] Hutto CJ, Gilbert EE. (2014). VADER: A Parsimonious Rule-based Model for Sentiment Analysis of Social Media Text. Eighth International Conference on Weblogs and Social Media (ICWSM-14). Ann Arbor, MI, June 2014.

[15] https://www.nltk.org/api/nltk.sentiment.SentimentIntensityAnalyzer.html?highlight=sentimentintensity.

[16] Calderon P. (2018). Vader Sentiment Analysis Explained. *Medium. 3*1 Mar. 2018, medium.com/@piocalderon/vader-sentiment-analysis-explained-f1c4f9101cd9.

[17] https://pypi.org/project/wordcloud/.

[18] https://cran.r-project.org/web/packages/nnet/index.html.

[19] Argueza BR, Saenz SR, McBride D (2021). From diversity and inclusion to Antiracism in Medical Training Institutions. Acad Med.

[20] https://pubmed.ncbi.nlm.nih.gov/35070089/.

[21] Yoder SR, Lonstein AB, Sharma A, Garcia-Munoz J, Moreno R, Chen AY, Orben G, Clemons T, Masters M, Forrest LL, Ukhuedoba I, Hall JM. PEARLS (Perspectives on Equity Advancement: Research and Learning Symposium), a Case Report in Promoting DEI in a Medical School Setting. *Education Sciences*. 2022; 12(9):586. 10.3390/educsci12090586.

[22] Leep Hunderfund AN, Reed DA, Starr SR, Havyer RD, Lang TR, Norby SM. Ways to Write a Milestone: Approaches to Operationalizing the Development of Competence in Graduate Medical Education. Acad Med. 2017;92(9):1328–1334. 10.1097/ACM.0000000000001660. PMID: 28353504. 10.1097/ACM.0000000000001660 28353504

[23] Tuma F. Nassar Ak. Feedback in Medical Education. [Updated 2022 Sep 26]. In: StatPearls [Internet]. Treasure Island (FL): StatPearls Publishing; 2022 Jan-. Available from: https://www.ncbi.nlm.nih.gov/books/NBK544311/. 31335031

[24] Burgess A, van Diggele C, Roberts C, Mellis C (2020). Feedback in the clinical setting. BMC Med Educ.

[25] Minor S, Bonnin R (2022). What do medical students want from a Mentor?. PRiMER.

[26] Lerchenfeldt S, Taylor TAH (2020). Best practices in peer Assessment: Training Tomorrow’s Physicians to obtain and provide Quality Feedback. Adv Med Educ Pract.

[27] Hardavella G, Aamli-Gaagnat A, Saad N, Rousalova I, Sreter KB (2017). How to give and receive feedback effectively. Breathe (Sheffield England).

[28] Leary JC, Schainker EG, Leyenaar JK (2016). The unwritten rules of mentorship: facilitators of and barriers to effective mentorship in Pediatric Hospital Medicine. Hosp Pediatr.

[29] Karadag E (2021). Effect of COVID-19 pandemic on grade inflation in higher education in Turkey. PLoS ONE.

[30] Andersen S, Leon G, Patel D, Lee C, Simanton E (2022). The impact of COVID-19 on academic performance and personal experience among First-Year Medical Students. Med Sci Educ.

