# Supplementary material for: Evaluation of bias and gender/racial concordance based on sentiment analysis of narrative evaluations of clinical clerkships using natural language processing
Source: BMC Med Educ. 2024 Mar 15;24:295. doi: 10.1186/s12909-024-05271-y (PMC10944013; doi:10.1186/s12909-024-05271-y)
Supplement: Supplementary file 1 — Supplementary Material 1 [file 12909_2024_5271_MOESM1_ESM.docx]

**Supplemental Tables**


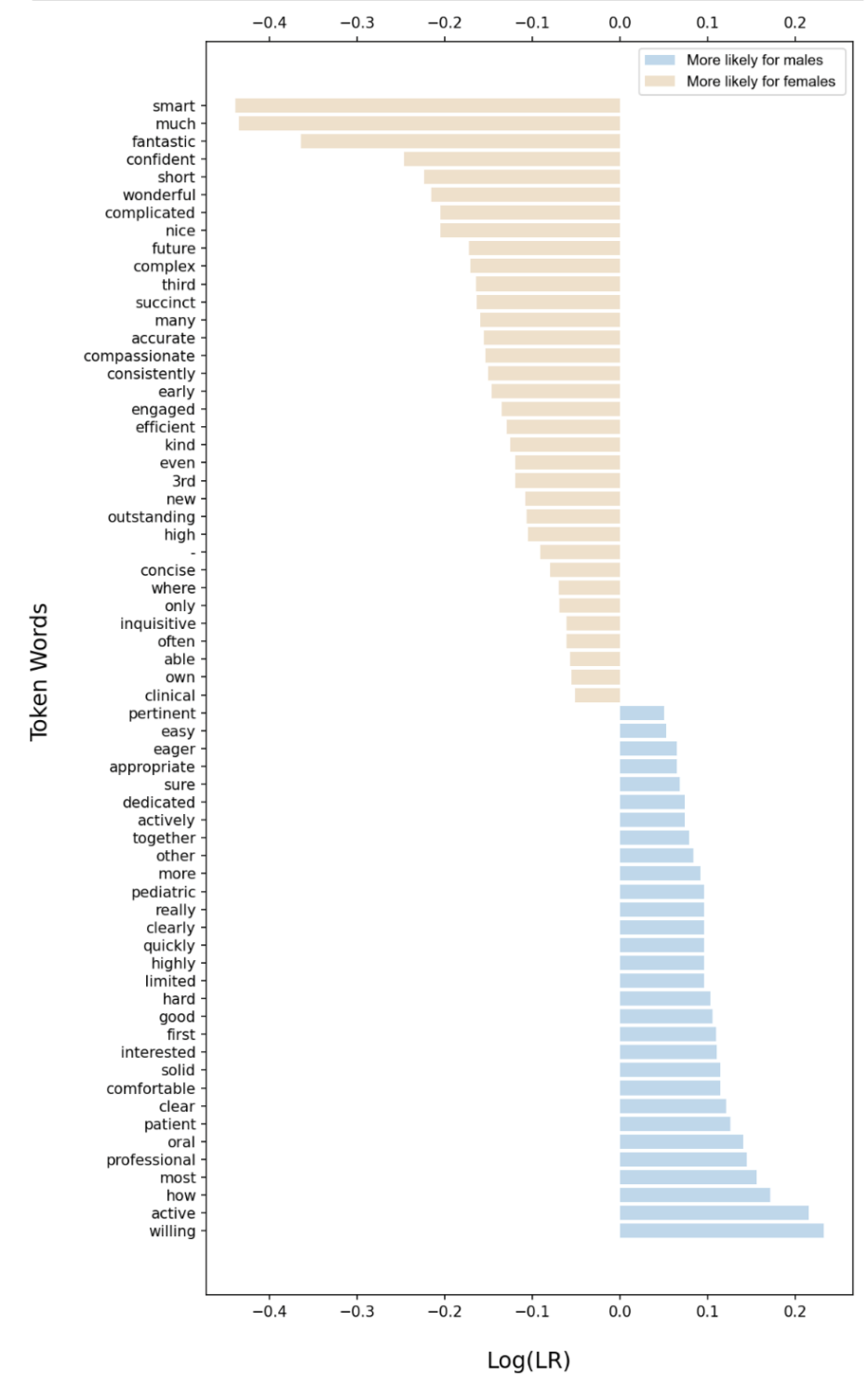


Supplemental Figure 1. Likelihood Ratios (LR) of token words used more likely for males versus females.


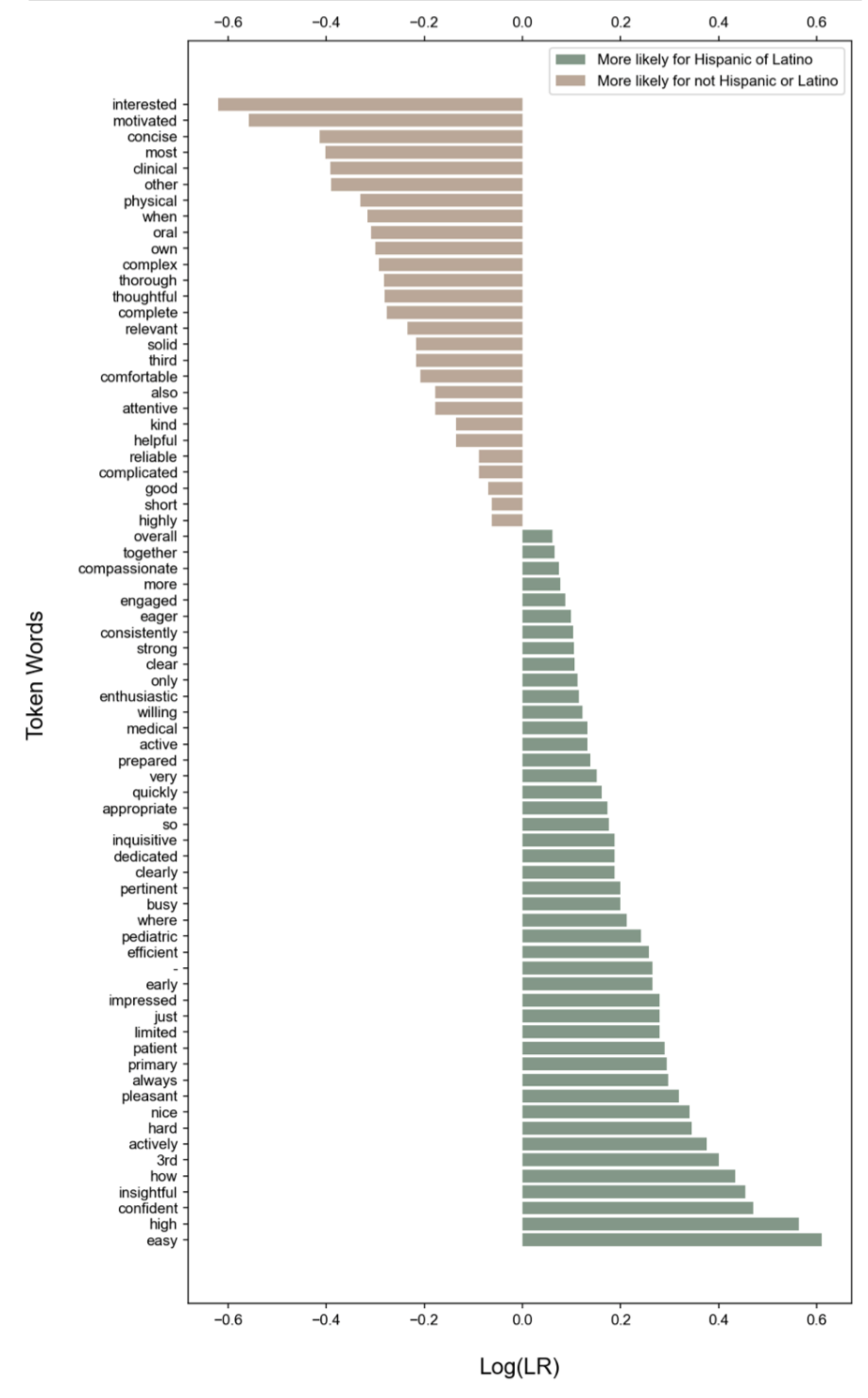


Supplemental Figure 2. Likelihood Ratios (LR) of token words used more likely for Hispanics/Latinos versus non-Hispanic/Latinos.


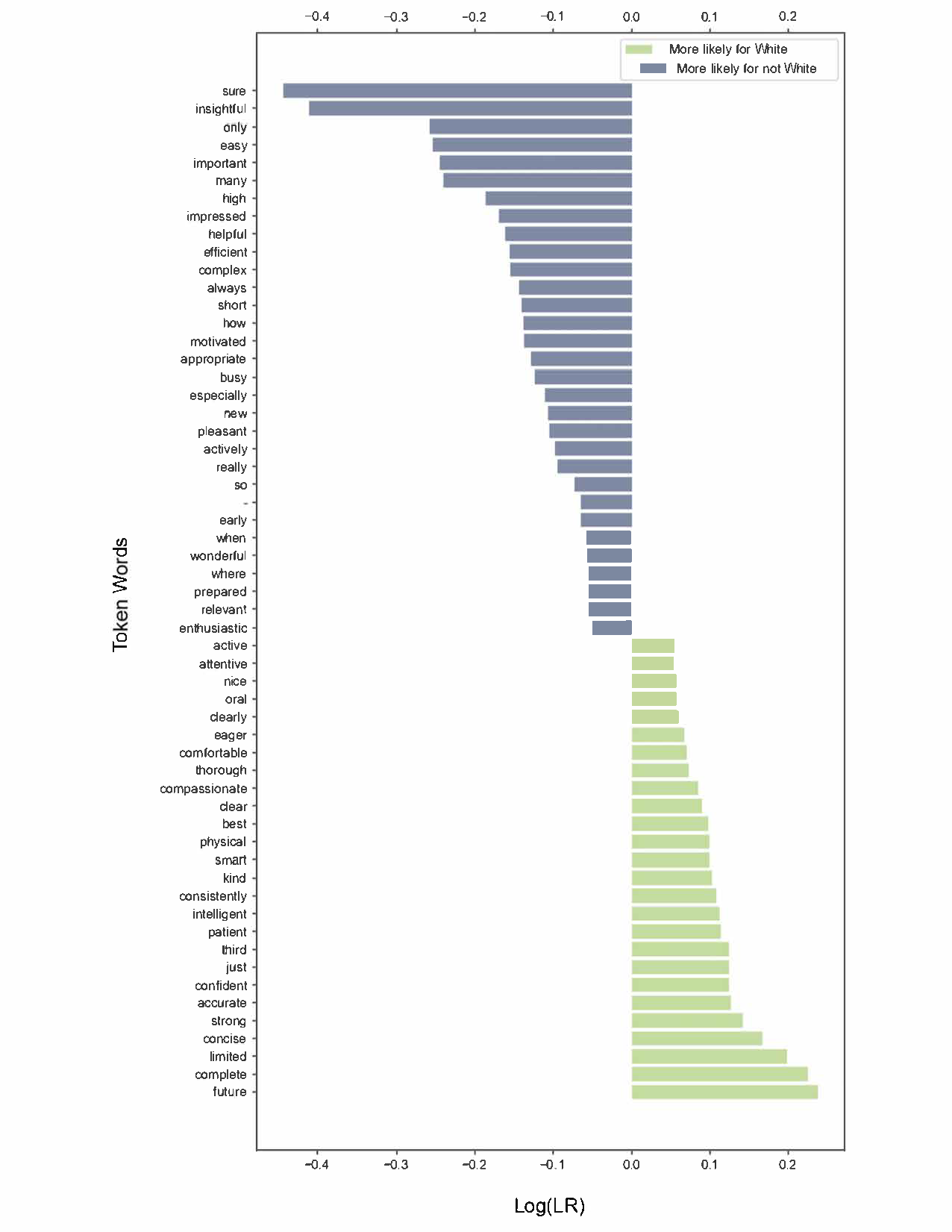


Supplemental Figure 3. Likelihood Ratios (LR) of token words used more likely for White students versus all other race groups.


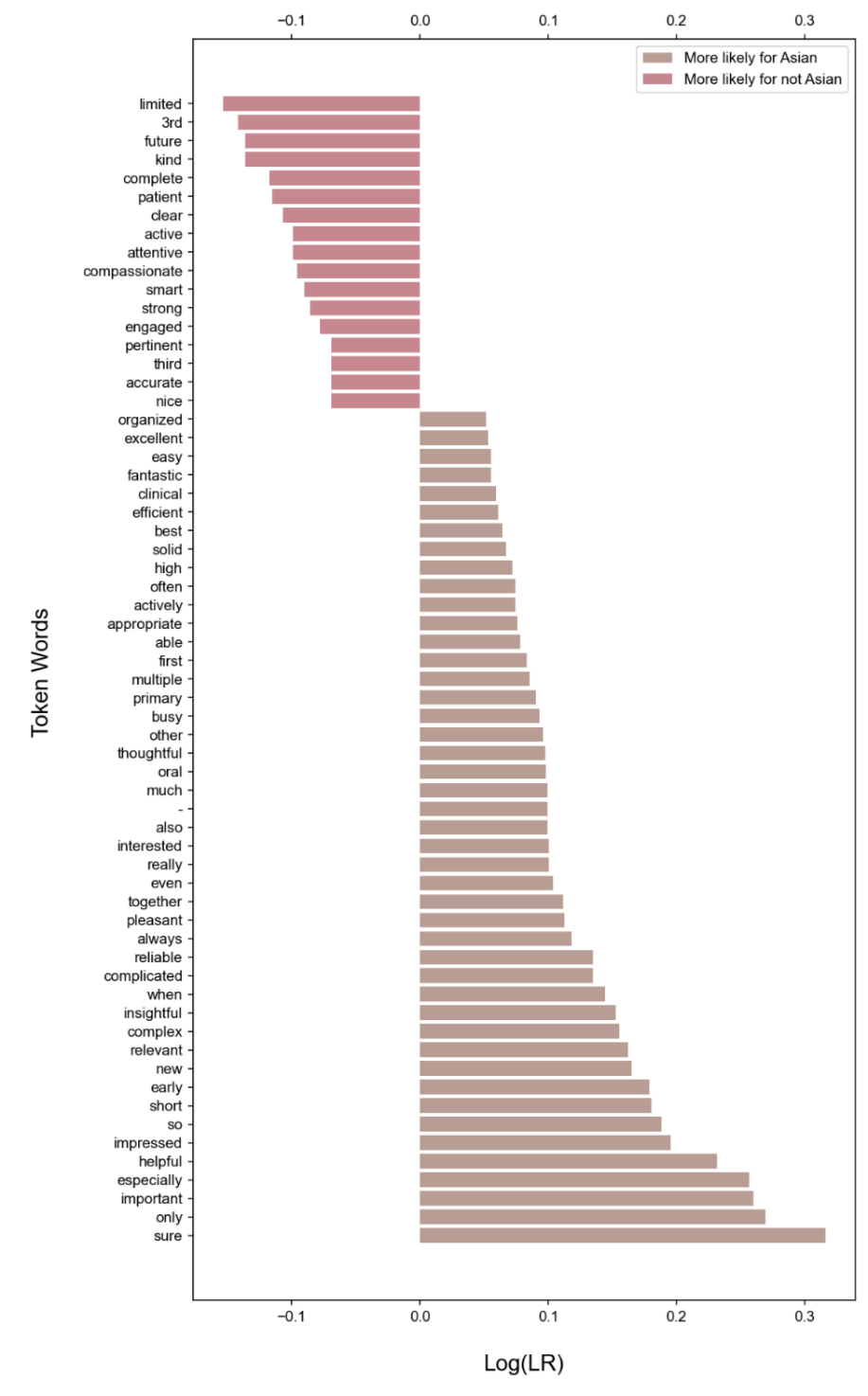


Supplemental Figure 4. Likelihood Ratios (LR) of token words used more likely for Asians versus all other race groups.

Supplemental Table 1. Sample reviewer comments with corresponding average evaluation scores (*identifiers are redacted).

| Reviewer Comments | Average Evaluation Score |
| --- | --- |
| [redacted] performed very well during his IM rotation at [redacted]. For [redacted] first rotation of his MS3 year, I felt he excelled compared to his peers in ability to start to formulate plans and write notes. On evaluation of one of his discharge summaries, he showed a good grasp of a patient's hospital course and was able to highlight important aspects of the patient's care. | 4.43 |
| [redacted] was a wonderful asset to the team during his rotation on our service. He had great enthusiasm, dedication, and caring. His bedside skills of data gathering were excellent to outstanding, he was thoughtful in developing his assessments and plans and enjoyed learning from every member of the team. He interacted well with staff and patients alike, professionally and with a smile. He carried 2 to 4 patients, which had multiple medical problems including one particular patient who was among the sickest on our team. [redacted] was also helpful in translating Spanish with some of our patients on bedside rounds. I believe he has great potential in his future as an internist or in whatever field he chooses. | 6.43 |
| [redacted] worked hard during his week on service with me. He was motivated, energetic, and always involved in the care of his patients and showed genuine interest in learning from all patients on the team. His written notes demonstrated depth of thought and reasoning that was discussed during rounds, though he required assistance with assimilation and assessment of clinical data which is to be expected at the beginning of the MS3 year. He has motivation to improve himself in regards to clinical reasoning and assessments and I think he will make improvements rapidly. | 3.00 |
| [redacted] was a hardworking, strong medical student. She was a great data-gatherer and synthesizer of information. She obtained a thorough history from all of her patients and picked up information that we missed. She was a positive contribution to her team, and was always enthusiastic to see new patients. | 7.00 |
| [redacted] demonstrated an average performance as a medical student. He was engaging and respectful, and will do well in whatever field he chooses | 3.86 |
